# Developing and piloting a self-assessment tool for medication review competence of practicing pharmacists based on nationally set competence criteria

**DOI:** 10.1186/s12913-021-07291-6

**Published:** 2021-11-25

**Authors:** Noora Lias, Tanja Lindholm, Marika Pohjanoksa-Mäntylä, Aleksi Westerholm, Marja Airaksinen

**Affiliations:** Clinical Pharmacy Group, Division of Pharmacology and Pharmacotherapy, Faculty of Pharmacy, University of Helsinki, Viikinkaari 5 E, P.O. box 56, 00014 Helsinki, Finland

**Keywords:** Medication review, Professional competence, Self-assessment tool, Professional development, Continuing education, Community pharmacy, Patient centeredness, Medicines policy

## Abstract

**Background:**

New competence requirements have emerged for pharmacists as a result of changing societal needs towards more patient-centred practices. Today, medication review competence can be considered as basic pharmaceutical competence. Medication review specific competence criteria and tools for self-assessing the competence are essential in building competences and a shared understanding of medication reviews as a collaborative practice. The aim of this study was to develop and pilot a self-assessment tool for medication review competence among practicing pharmacists in Finland.

**Methods:**

The development of the self-assessment tool was based on the national medication review competence criteria for pharmacists established in Finland in 2017 and piloting the tool among practicing pharmacists in a national online survey in October 2018. The pharmacists self-assessed their medication review competence with a five-point Likert scale ranging from 1 for “very poor/not at all” to 5 for “very good”.

**Results:**

The internal consistency of the self-assessment tool was high as the range of the competence areas’ Cronbach’s alpha was 0.953–0.973. The competence areas consisted of prescription review competence (20 items, Cronbach’s alpha 0.953), additional statements for medication review competence (11 additional items, Cronbach’s alpha 0.963) and medication review as a whole, including both the statements of prescription review and medication review competence (31 items, Cronbach’s alpha 0.973). Competence items closely related to routine dispensing were most commonly self-estimated to be mastered by the practicing pharmacists who responded (*n* = 344), while the more clinical and patient-centred competence items had the lowest self-estimates. This indicates that the self-assessment tool works logically and differentiates pharmacists according to competence. The self-assessed medication review competence was at a very good or good level among more than half (55%) of the respondents (*n* = 344).

**Conclusion:**

A self-assessment tool for medication review competence was developed and validated. The piloted self-assessment tool can be used for regular evaluation of practicing pharmacists’ medication review competence which is becoming an increasingly important basis for their contribution to patient care and society.

## Introduction

Pharmacists are among the professions that face the challenge of developing their competences to meet the changing needs of society. For pharmacists, this means shifting in a more clinical and patient-centred direction to improve the effectiveness of pharmacotherapies and reduce preventable drug-related morbidity and mortality rates [1–3]. This trend is reinforced by aging populations worldwide that set new competence requirements for pharmacists. As a result, there has been a global call for community pharmacists to take more responsibility for patient care by reviewing medications for individual patients [1, 4–6]. This enables identifying, solving and even preventing therapeutically significant medication-related problems and risks that are found to be common, especially in older adults [7–17].

Medication review competence can be considered as a contemporary, basic pharmaceutical competence that should be acquired in undergraduate training [18, 19]. Even so, there are a high number of practicing pharmacists who lack these crucial competences, or at least need to improve their current skills [4, 20]. Several national and international general level competence frameworks have been developed for pharmacists [21–30]. However, no specific medication review competence criteria nor tools for self-assessing medication review competence have been published so far, although these may be important facilitators for learning. Harmonised concepts and defined contents also can enable building a shared understanding of medication reviews as a collaborative practice. The aim of this study was to develop and pilot a self-assessment tool for medication review competence based on nationally set competence criteria.

## Methods

### Context

This study was conducted in Finland, where community pharmacies are the sole source of prescription and non-prescription medicines in outpatient care [31, 32]. Since 1983, community pharmacists have been obliged to ensure the safe and appropriate use of medicines by counselling while dispensing. Consequently, medication counselling has become an essential task, which is continuously supported by medicine policy initiatives and national development strategies targeting community pharmacy functions [33, 34]. Nowadays community pharmacies are considered one of the most important medicines information sources [35, 36]. However, compared to the shift in tasks, the development of community pharmacists’ competence has been lagging behind, even though undergraduate and continuing education have evolved remarkably [4, 20].

While communication on medication was enhanced in pharmacies, it became evident that not all medication-related problems can be solved by counselling [37–40]. Therefore, a collaborative, comprehensive medication review procedure was developed, and related accreditation training was initiated in Finland in 2005 [41, 42]. The need for specific medication review competence criteria became evident in 2014 when medication review competence was integrated into the pharmacy undergraduate curriculum first at the University of Helsinki as part of the curriculum reform and later in other pharmacy schools [18, 19]. As no previous competence criteria were found even internationally, The National Coordination Group AATE consisting of key stakeholders in the community pharmacy field started the development process and published the criteria for pharmacist-conducted prescription reviews, medication reviews and comprehensive medication reviews in 2017 [43].

### Development of medication review competence criteria

The development process began by describing the basic features of pharmacist-conducted prescription reviews, medication reviews and comprehensive medication reviews by using the categorisation by the National Health Service (NHS) in the UK [44]. Their categorisation was modified to fit the Finnish context and legislation while taking into account the Finnish Medicines Agency’s guidelines on optimising pharmacotherapy for older adults [45], the Medicines Policy 2020 by the Ministry of Social Affairs and Health [33], existing national medication review definitions [46] national and international research on collaborative medication review practices [13, 16, 41, 47–53] and current clinical pharmacy and medication review related undergraduate and continuing education [19].

The competence criteria were developed using a consensus method [43]. The work was led by a working group of medication review experts under the national AATE Coordination Group (later AATE). The working group was responsible for the development rounds and the revision of the draft competence criteria according to the feedback received between the rounds. Five rounds were needed to achieve consensus. Members of the working group and other invited medication review experts acted as an expert panel. Feedback on the draft competence criteria was also received between the rounds from 1) other members of AATE (three rounds of comments), 2) from comprehensive medication review accredited pharmacists (*n* = 30) and 3) from three physicians. Final approval was received from AATE. The final competence criteria included 17 items for prescription review, 10 additional items for medication review and three additional items for comprehensive medication review.

### Development of the self-assessment tool

**Fig. 1:**
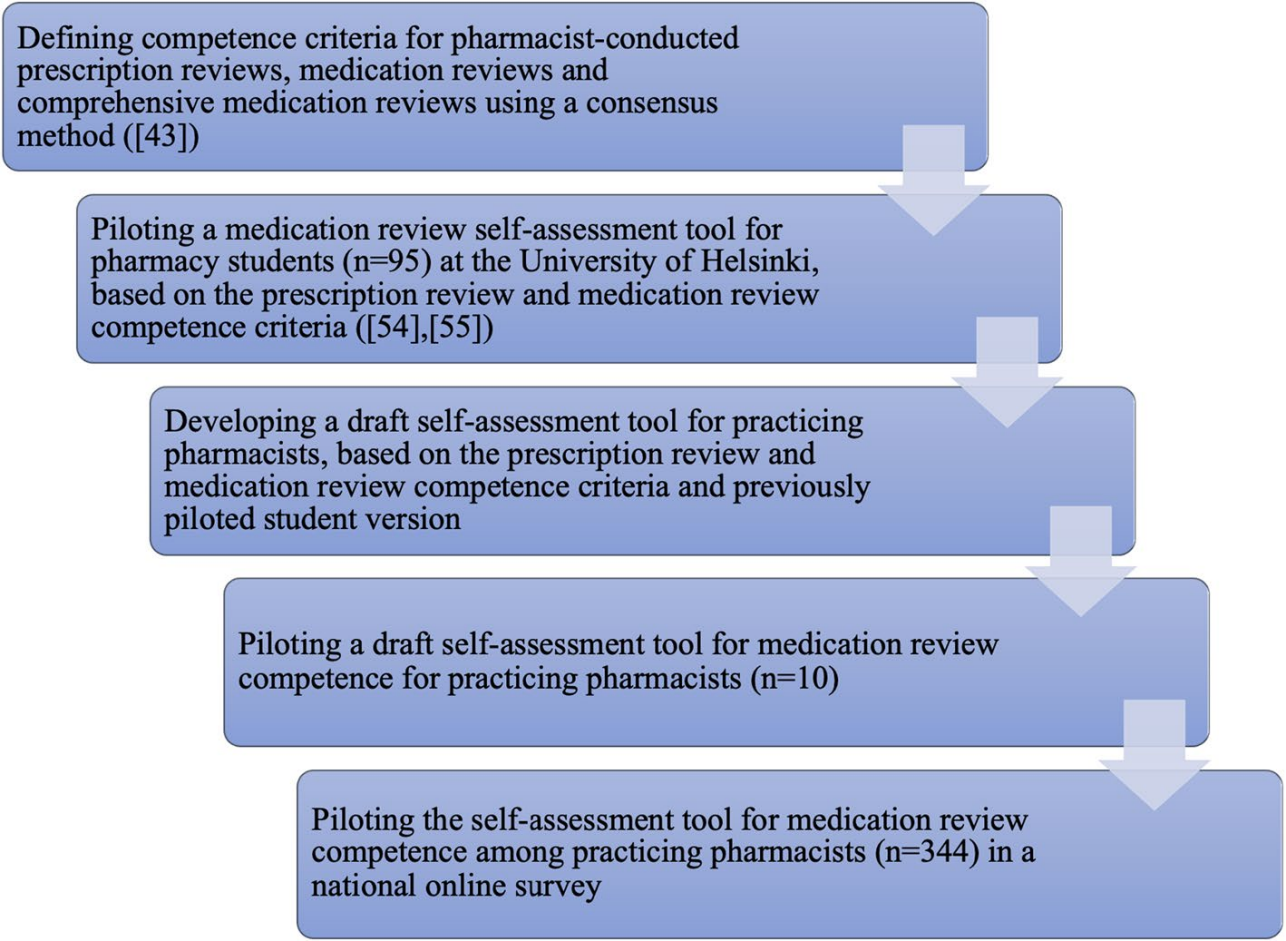
The development and validation process of the self-assessment tool for medication review competence for practicing pharmacists based on nationally set competence criteria

**Fig. 2:**
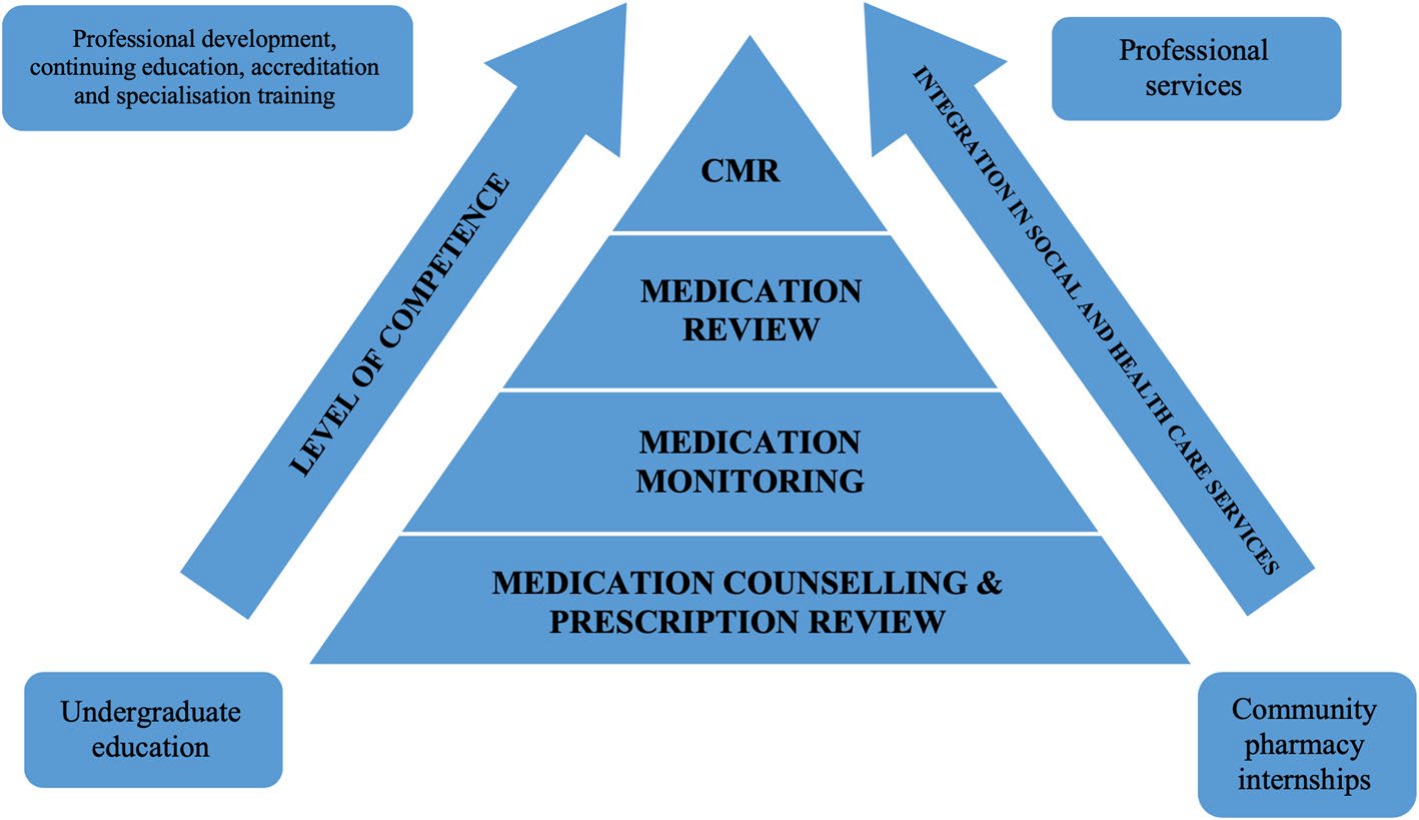
Illustration of competence development from competence needed in medication counselling towards competence needed in monitoring and reviewing medications [43, 55]. CMR = Comprehensive medication review requiring accreditation training

The self-assessment tool used in the study was based on the prescription review and medication review competence criteria established by AATE in 2017 (Fig. 1) [43]. At first, the self-assessment tool was piloted among third year pharmacy students at the University of Helsinki in 2017–2018 [54, 55]. This student version was used in the development of the next version of the self-assessment tool. The next version was piloted with a convenience sample of 10 practicing pharmacists during the summer 2018. Based on their feedback, one prescription review related statement was divided into three individual statements and one statement into two. Three of the newly-formed statements were not included in the original AATE competence criteria. These statements were considered necessary regarding medication review competence and were added to the survey by the judgement of the research group. The decision was supported by the working group operating under AATE [43]. Thus, the tool included a total of 31 competence criteria, of which 20 were for the prescription review competence, and the additional 11 were for acquiring medication review competence. The pharmacists were instructed to self-assess their competence with a 5-point Likert scale ranging from 1 for “very poor/not at all” to 5 for “very good”. The statements were arranged in an ascending order based on the required competence level (Fig. 2). The competence criteria for prescription reviews came first and then the additional competence criteria for medication reviews.

### Data collection

The data was collected by using an electronic survey targeted to all graduated practicing pharmacists belonging to The Finnish Pharmacists’ Association or The Finnish Pharmacists’ Society. Pharmacy students were excluded from the survey. There are approximately 9000 pharmacists in Finland, the majority being covered by these two national professional associations. The cover letter for the survey and the link to submit responses electronically were sent through The Finnish Pharmacists’ Association to pharmacists who were on their membership registers in October 2018. The Finnish Pharmacists’ Society informed its members about the survey via the society’s intranet. Two reminders were sent via the associations and the deadline for responses was extended in order to receive more responses.

### Data analysis

The internal consistency of the self-assessment tool was defined using a reliability analysis (Cronbach’s alpha).

Data from the pilot self-assessment survey was analysed for descriptive statistics using Microsoft Excel (version 2016) and was presented as frequencies, percentages and summative scales. Two figures were compiled from Likert scale statements describing self-assessed competence in prescription review (20 statements) and medication review (11 additional statements). Two sum variables were also formed: 1) for the self-assessed competence in prescription review (20 statements) and 2) for the self-assessed competence in medication review as a whole including both the competence criteria for prescription review and the additional competence criteria for medication review (20 + 11 statements).

The highest combined “good” and “very good” self-estimates were determined to represent the best competence, whereas the highest combined “very poor/not at all” and “poor” responses were determined to represent the poorest competence. The summative variable for prescription review and medication review competence was formed by giving the respondent a competence score ranging from 1 to 5, depending on the self-assessment score level, for each of the individual competence criteria (Table 1). Thus, the competence score ranged from 20 to 100 for the 20 prescription review statements and from 31 to 155 for the 31 medication review statements. Grade 1 represented the poorest competence and the grade 5 the highest competence.

**Table 1:** Score limits when forming the summative variables for prescription review (*n* = 20) and medication review (*n* = 31) competences. The score limits were formed by dividing the score range by five. Competence scores were categorised using 5 grades so that grade 1 represented very poor competence, grade 2 poor competence, grade 3 moderate competence, grade 4 good competence and grade 5 very good competence

Grade	Prescription review	Medication review
1	20–35	31–55
2	36–51	56–80
3	52–68	81–105
4	69–84	106–130
5	85–100	131–155

## Results

The internal consistency was high for the statements measuring prescription review competence (20 statements, Cronbach’s alpha 0.953), medication review competence (11 additional statements, Cronbach’s alpha 0.963) and medication review competence as a whole (31 statements Cronbach’s alpha 0.973) (Table 2).

**Table 2:** The internal consistency of the competence statements for prescription review and medication review (Cronbach’s alpha)

Level of review	Number of items (n)	Cronbach’s alpha
Prescription review	20	0.953
Medication review (additional statements)	11	0.963
Medication review (as a whole, a sum variable)	31	0.973

By the end of October 2018, a total of 344 practicing pharmacists had responded after extending the deadline for responses and sending two reminders. Most of the respondents (94%) were female, had a BSc (Pharm) degree (73%), worked in a community pharmacy (64%) and had at least 10 years of work experience (65%) (Table 3).

**Table 3:** Characteristics of the respondents (*n* = 344)

Variable	%	n
**Gender**		
Female	94	325
Male	6	19
**Degree**^**a**^		
BSc (Pharm)	73	253
MSc (Pharm)	24	82
PhD (Pharm)	3	9
**Previous degree (not pharmacy related)**		
No previous degree	66	228
BSc, MSc or PhD at a university or a university of applied sciences/vocational education and training		
Health care	13	46
Non-health care	13	44
Other	8	26
**Current workplace**		
Community pharmacy	64	221
Primary or secondary care unit	21	73
Other	12	40
Staff leasing company	3	10
**Work experience as a pharmacist, years**		
0–3	13	46
4–9	22	76
10–15	22	74
> 15	43	148
**Completed long-term continuing or accreditation trainings**^**b**^		
Prescription review^c^	26	89
Medication review^d^	16	55
Comprehensive medication review^e^	13	44
Specialisation in ward pharmacy^f^	3	9
Specialisation in community pharmacy^ g^	5	16

^a^The university education program in Finland for pharmacists consists of two tiers: the BSc (3 years, 180 ECTS credits) and MSc (5 years, 180 + 120 ECTS credits) curriculums, ECTS: European Credit Transfer System

^b^The respondents were able to choose several alternatives

^c^Continuing training offered by the Pharmacy Learning Centre

^d^1-year continuing training (20 ECTS credits) or undergraduate studies starting from 2014 at the University of Helsinki, followed by other pharmacy schools in 2017

^e^1.5 Years’ accreditation training (35 ECTS credits)

^f^Accreditation training (30 ECTS credits)

^g^Post-graduate programme: BSc (Pharm) 3-year specialisation studies (40 ECTS), MSc (Pharm) 4-year specialisation studies (60 ECTS)

### Prescription review competence

**Fig. 3:**
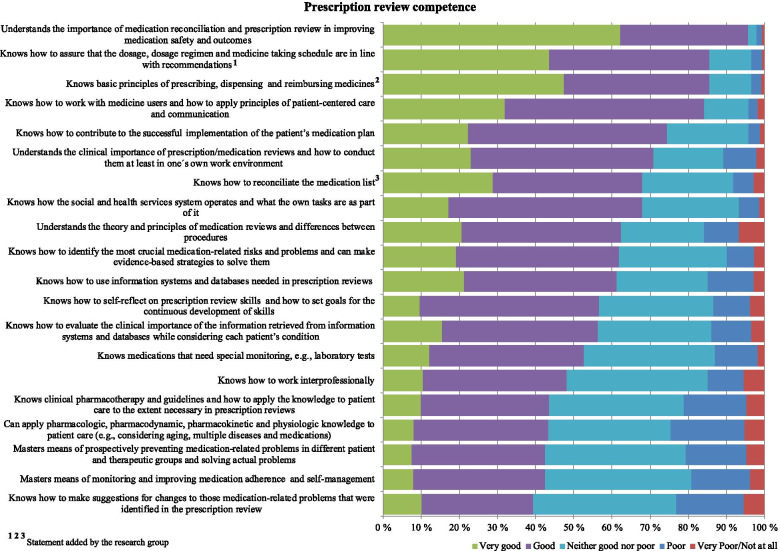
Self-assessed prescription review competence for practicing pharmacists (% of the respondents, *n* = 344). The internal consistency of the statements (*n* = 20) in the reliability analysis was 0.953 (Cronbach’s alpha)

The internal consistency of the statements measuring prescription review competence (*n* = 20) was high (Cronbach’s alpha 0.953) (Table 2). The highest combined “good” and “very good” self-estimates for the 20 competence areas required for conducting prescription reviews were obtained for the following three statements: 1) Understands the importance of medication reconciliation and prescription review in improving medication safety and outcomes (96%), 2) Knows how to assure that the dosage, dosage regimen and medicine taking schedule are in line with recommendations (85%), and 3) Knows basic principles of prescribing, dispensing and reimbursing medicines (85%) (Fig. 3).

Respectively, the highest combined “very poor/not at all” and “poor” scores were obtained for the following three statements: 1) Can apply pharmacologic, pharmacodynamic, pharmacokinetic and physiologic knowledge to patient care (e.g., considering aging, multiple diseases and medications) (25%), 2) Knows how to make suggestions for changes to those medication-related problems that were identified in the prescription review (23%), and 3) Knows clinical pharmacotherapy and guidelines and how to apply the knowledge to patient care to the extent necessary in prescription reviews (21%) (Fig. 3).

### Medication review competence

**Fig. 4:**
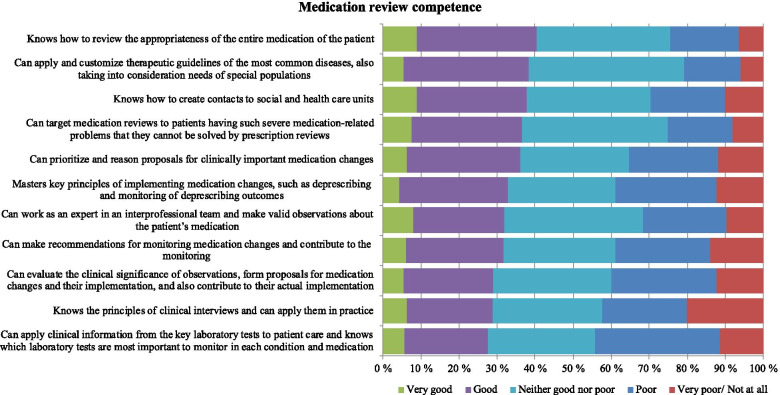
Self-assessed medication review competence of the practicing pharmacists (% of the pharmacists, *n* = 344). The internal consistency of the statements (*n* = 11) in the reliability analysis was 0.963 (Cronbach’s alpha)

The internal consistency of the 11 additional statements measuring medication review competence was high (Cronbach’s alpha 0.963) (Table 2). The highest combined “good” and “very good” self-estimates for the 11 competence areas required for conducting medication reviews were obtained in the following three statements: 1) Knows how to review the appropriateness of the entire medication of the patient (40%), 2) Can apply and customize therapeutic guidelines of the most common diseases, also taking into consideration needs of special populations (38%), and 3) Knows how to create contacts to social and health care units (38%) (Fig. 4).

The highest combined” very poor/not at all” and “poor” shares for the competence areas required for conducting medication reviews were obtained for the following three statements: 1) Can apply clinical information from key laboratory tests to patient care and knows which laboratory tests are most important to monitor in each condition and medication (44%), 2) Knows the principles of clinical interviews and can apply them in practice (42%), and 3) Can evaluate the clinical significance of observations, form proposals for medication changes and their implementation, and also contribute to their actual implementation (40%) (Fig. 4).

### The summative competence scores in conducting prescription reviews and medication reviews

**Fig. 5:**
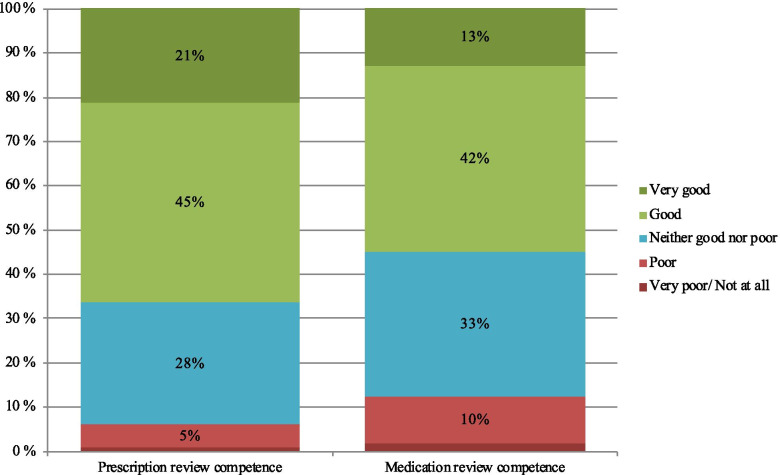
The summative competence for prescription reviews (20 competence statements) and medication reviews (31 competence statements) based on a self-assessment of practicing pharmacists (*n* = 344)

Among the respondents, 66% reached the grade “very good” (21%) or “good” (45%) when counting the summative self-assessed competence scores in conducting prescription reviews. For 6% of respondents, their self-assessed prescription review competence was graded as “very poor/not at all” or “poor” based on the summative competence scores (Fig. 5). Among the respondents, 55% reached the grade “very good” (13%) or “good” (42%) when the summative self-assessed competence scores in conducting medications reviews were counted. Twelve percent of the respondents’ self-assessed medication review competence was graded as “very poor/not at all” or “poor” based on the summative competence scores (Fig. 5).

### Future prospects of medication review education

The majority of the respondents (80%), who did not possess medication review expertise at the time of the study, were willing to complete or verify this in the future. Online-education (39%) and long-term continuing education (30%) were the preferred methods. Six percent had chosen the “Other, please specify” -alternative and suggested, for example, a multi-modal course, combining online and on-the-job training.

## Discussion

The self-assessment tool for medication review competence can be considered valid and able to distinguish different levels of competence as the Cronbach’s alpha value is > 0.70 at all points ([56, 57], Table 2). The results for prescription review and medication review were logical, i.e., self-assessed prescription review expertise was at a higher level than medication review expertise that requires a higher level of competence.

To our knowledge, this was the first attempt to develop a tool measuring medication review competence of pharmacists as no previous tool or medication review specific competence criteria were found in an extensive international inventory during the development process of the national competence criteria [43]. The competence criteria for prescription and medication review that functioned as the self-assessment tool’s basis were thoroughly and rigorously developed by national experts in the area during a multistage process. Since then, the tool has been piloted by both pharmacy students and practicing pharmacists and modified based on the feedback [43, 54, 55]. In the absence of equivalent self-assessment tools to compare with, the researchers applied a scoring system based on a five-point Likert scale, which is frequently used in self-assessment studies, to measure the competence for each item and to compose the sum variables. Thus, the self-assessment tool’s scores with grades ranging from 1 to 5 were considered to reflect the competence levels of the pharmacists. Most of the existing competence frameworks for pharmacists are very broad and, therefore, represent massive entities that remain at a general level [21–30]. It would be useful to fine-tune the frameworks and make them more focused and detailed, even split them into several specialty areas, one of them being medication review competences. This would also raise awareness of what the competence requirements of pharmacy professionals are and which competences should be developed [58]. Hopefully, the self-assessment tool that was created in this study will raise discussion and undergo further development that will enable its utilization in national and international pharmacy practice contexts.

Based on the findings of the pilot study, the self-assessed medication review competence was at a very good or good level among more than half (55%) of the practicing pharmacists who responded (Fig. 5). In general, it can be noticed that the “very good” proportions decreased sharply when moving from the competence criteria for prescription review to the competence criteria for medication review. Correspondingly, the “very poor/not at all” proportions increased when moving from the competence criteria for prescription review to the competence criteria for medication review. This reflects a more comprehensive management of prescription review related skills compared to medication review related skills. This is in line with the fact that medication review expertise requires separate additional training or completion of the BSc (Pharm) degree by following the renewed undergraduate curriculum (started in 2014) [18].

According to a previous self-assessment study, third-year pharmacy students at the University of Helsinki who had attended the reformed BSc (Pharm) curriculum, had a higher level of self-assessed medication review competence than the practicing pharmacists participating in this study [54, 55]. This may indicate that the integration of medication review competence into the BSc (Pharm) curriculum has achieved the set goals of developing the competences towards a clinical and patient-centred direction. Previous international studies have made similar observations about the better self-assessed competence of the younger generation, but also about the growing need for postgraduate training and the mismatch or outright lack of its provision, with prevailing and rapidly changing competence requirements in the field [58–60]. This, in turn, indicates the existence of intergenerational competence gaps, although it should be noted that our results may be affected by a low response rate and the Dunning-Kruger effect [61, 62]. Thus, more efforts need to be directed towards previously graduated practicing pharmacists to keep their competences updated.

Developing easy-to-use competence self-assessment tools for practicing pharmacists, such as the one developed in this study, could support the desired shift from drug-centred pharmacy practice towards a more patient-centred one by identifying competence gaps and educational needs [1, 63, 64]. The findings from this self-assessment pilot survey indicate that drug-centred competences related to dispensing were still the best mastered ones. The results are in line with previous international studies [65–67]. They may reflect the fact that the real-life pharmacy practice and education in Finland and elsewhere, have mainly focused on these drug-centred practices and competences.

In line with previous studies, shortcomings in applied clinical pharmacotherapy competences were implied [54, 55, 63, 65, 68, 69]. This concerns key competences needed for reviewing medications, such as estimating the clinical significance of the identified medication-related problems and risks, participating in implementing medication changes and interpreting and understanding laboratory test values. As the proportion of the aging population is growing, the importance of geriatric applied pharmacotherapy will also increase because of many age-related factors influencing drug suitability and dosing [20, 51, 70]. Furthermore, addressing the implied competence gaps related to interprofessional collaboration and collaboration with patients and their proxies could support medication adherence and self-management, also avoid preventable harm [4, 15, 20, 45, 52, 53, 71–74].

Additional education could increase pharmacists’ clinical competence and promote the implementation of pharmaceutical care and patient-centred practice [75–78]. According to this pilot study, practicing pharmacists are interested in developing, complementing and validating their medication review skills, primarily in the form of online education and long-term continuing education. In line with the results of this pilot survey, it can be generally noted that pharmacists want to stay informed about the advances of their profession and are willing to maintain and develop their professional competence to meet the needs of a changing society [63, 64, 79, 80].

### Strengths and limitations of the study

The internal consistency of the result descriptors and sum variables was defined using a reliability analysis (Cronbach’s alpha) (Table 2) [56, 57]. Cronbach’s alpha value was high as it was > 0.70 at all points. Content and face validity were supported by the facts that the competence criteria were developed by medication review experts in Finland and that the self-assessment tool was piloted among third year pharmacy students (*n* = 95) of the University of Helsinki, as well as among practicing pharmacists (*n* = 10; 5 with a BSc (Pharm) degree, 5 with a MSc (Pharm) degree) [43, 54, 55, 81]. Construct validity was supported by a high Cronbach’s alpha value and by the fact that the results of the survey were logical, i.e., the “very good” proportions decreased sharply when moving from the competence criteria for prescription review to the competence criteria for medication review. Similarly, the “very poor/not at all” proportions increased when moving from the competence criteria for prescription review to the competence criteria for medication review.

The number of respondents (*n* = 344) in this study was low but adequate for the purposes of a pilot validation study [57]. However, national generalisations of the results cannot be made based on this study. The survey had interested most practicing pharmacists with at least 15 years of work experience as 43% of the respondents were from this segment. They can be considered the main target group for efforts to maintain professional competence updated. It was not possible to calculate the response rate. This is because The Finnish Pharmacists’ Society informed its members about the survey via the Society’s intranet, so the exact number of the target population is not known. As the respondents were presumably interested in and familiar with the topic, with more in-depth knowledge the results may give an overly optimistic picture of medication review competences in general. The majority of the respondents worked in community pharmacies (64%) or in primary or secondary care (21%) in which collaborative medication reviews are supposed to be implemented and conducted [15, 16, 82–85]. Furthermore, the Dunning-Kruger effect relating to self-assessment, i.e., the bias of superiority where an individual overestimates his or her competence can be considered a limitation of the survey [61, 62].

### Practical implications

The self-assessment tool for medication review competence developed in the study can be used to identify medication review competence gaps and to monitor the development of the national implementation of medication review competence in the future. It can serve as a model when designing similar studies or tools internationally. The self-assessment tool can be used by practicing pharmacists when self-reflecting upon their competence and use the information gained, for example, in professional development discussions or when considering verifying the medication review competence through the portfolio-based verification process. The self-assessment tool for medication review competence has already been used as a basis for a self-assessment tool in the portfolio-based verification process of medication review competence in Finland. The study gave an insight into the competence gaps and educational needs of practicing pharmacists and the direction in which education and competence should be developed in the future.

### Further research

The development of the situation concerning medication review competence should be monitored periodically, for example, by conducting a self-assessment tool-based survey every 5 years. In this way, information on the implementation of the medication review competence in the pharmacy field is provided and the competence to work as part of healthcare is ensured. Further research is needed regarding the implementation of medication review practices, especially in the community pharmacy setting.

## Conclusions

A self-assessment tool for medication review competence was developed and validated. The results were logical, and the tool was found to be able to distinguish different levels of competence and reveal competence gaps. Activities related to dispensing were self-assessed at the best level. Educational needs were identified regarding patient-centred and clinical competence. The results are in line with previous international studies and reflect competence areas which have traditionally been considered a strong area of expertise in the pharmacy field. Continuing and postgraduate medication review training is required for pharmacists who have previously graduated to effectively implement medication review competence within the profession. The self-assessment tool for medication review competence can be used as a regular monitoring tool for evaluating practicing pharmacists’ medication review competence and directing continuing education.

## Data Availability

The datasets used and/or analysed during the current study are available from the corresponding author on reasonable request.
